# Factors influencing the financing behavior of large professional households engaged in green agricultural production in China

**DOI:** 10.3389/fpsyg.2022.820575

**Published:** 2023-01-10

**Authors:** Shen Zhong, Xueting Xu, Junwei Li, Nanlin Wu

**Affiliations:** ^1^School of Finance, Harbin University of Commerce, Harbin, Heilongjiang, China; ^2^School of Humanities, Social Sciences & Law, Harbin Institute of Technology, Harbin, Heilongjiang, China

**Keywords:** large professional households, green agricultural, theory of planned behavior, financing behavior, influence factor

## Abstract

Green agriculture is the direction of sustainable development of China’s modern agriculture and the inherent requirement of taking the road of characteristic new agricultural modernization. As one of the main bodies of new agricultural management, professional large households are of great significance to lead the development of efficient agriculture and accelerate the development of green agricultural economy in Heilongjiang. Therefore, based on the theory of planned behavior, this article includes 275 major professional households in Heilongjiang Province as a sample of demonstration counties (cities) for green, high-quality, and efficient creation, combined with field research data and structural equation model, and discusses the willingness of large professional households to participate in green agricultural production financing and behavioral factors and mechanisms of action. The perceived behavior control (PBC), attitude toward the behavior (AB), and subjective norms (SNs) of large professional households to engage in green agricultural production determine their financing intentions (FNs), which further determines their financing behavior (FB). The results show that PBC, AB, and SN have a significant positive impact on the FN of large professional households, and further indirectly affect the FB. It should be noted that SNs have the most significant impact on the FN of large professional households to participate in green agricultural production. Therefore, it is necessary to establish effective government propaganda measures and preferential policies, improve the awareness of the importance of green agricultural production financing, and create a good social atmosphere for agricultural sustainable development and active participation in financing. The purpose of this study is to provide a reference for policymakers to formulate relevant policies to cultivate major professional households and develop green agricultural economy in Heilongjiang Province.

## 1. Introduction

Green agriculture is an inevitable requirement of China’s agricultural economic development. China’s agricultural development has made great achievements; however, the long-term extensive mode of production and the neglect of the ecological environment lead to the increasingly prominent contradiction between agricultural production and the ecological environment. The soil conditions of agricultural production are superior. Heilongjiang Province is the largest plain in China’s black land and one of the three black land regional plains in the world. The soil’s organic matter is very rich. The output of green agriculture in Heilongjiang Province ranks first in China and is an important base for food security in China.

As an important agricultural base in China, although Heilongjiang has certain resource advantages, it also faces the dual pressure of resource conditions and ecological environment in recent years. Modern agriculture has higher productivity than traditional agriculture, but it poses a threat to resources and the environment. Therefore, the development of green agricultural production is consistent with the goal of ecological agriculture, which plays a role in protecting the environment of producing areas and ensuring the sustainable development of agriculture (Ali et al., 2015). The development of green agriculture is based on ecological agriculture, which is a scientific and green development concept (Carneiro et al., 2017). Koohafkan (2012) believes that the development of green agricultural production can scientifically and effectively adjust the allocation structure of agricultural production factors, ensure the safe supply of agricultural products, and optimize the ecological environment. Therefore, the development of green agricultural production is of great significance for the industrialization and sustainable development of green agriculture in Heilongjiang Province.

The development of green agriculture emphasizes the conservation and efficient utilization of resources. It needs sufficient funds to improve the traditional production mode through technological innovation. Driven by the marketization of the rural economy and the diversification of farmers’ operation, farmers’ demand for funds is increasing (Sijtsma and Molenaar, 2002). However, the lack of funds has become the most important obstacle to the development of green agricultural production. Green agricultural production integrates all links before, during, and after production, and promotes the integration of business mode, socialization of service, and enterprise management (Hattam and Holloway, 2005). Due to the lack of funds for most farmers, farmers continue to use traditional production methods in agricultural production, which affects the enthusiasm of farmers to participate in green agricultural production (Ingram, 2010). It is undeniable that farmers have sufficient funds for green agricultural production, which is the premise of green agricultural development. Therefore, the development of green agriculture requires a lot of financial support from the government.

With the rigid growth in domestic demand for agricultural products and the continuous improvement in quality and safety requirements, the agricultural production mode characterized by small farmers’ intensive cultivation is challenged, and it is crucial to speed up the cultivation of new agricultural operators (Banjongrewadee et al., 2020). In recent years, new agricultural entities such as professional large households, family farms, and farmers’ cooperatives can organize scattered farmers to carry out green agricultural production, develop innovative green agricultural production mode, ensure the quality and safety of agricultural products, and promote the development of regional green agricultural industrialization (Durak and Saritepeci, 2017). As one of the main bodies of new agricultural management, with the continuous increase in land scale and policy support in China, the number of professional large households has increased greatly and has provided an important force for green agricultural production. Large professional households have moderate-scale and specialized production characteristics and have a good sense of modern operation and management (Lohr and Salomonsson, 2000). However, from the perspective of personal characteristics, due to the influence of age, education level, risk preference, and environmental protection concept, large professional households have a certain cognitive bias to engage in green agricultural production and financing. This will further affect the scale and quality of green agricultural production (Yuelai et al., 2020). Therefore, the willingness and behavior of green agricultural production financing of large professional households is the key to realizing green professional and sustainable development of agriculture in China.

At present, the above research mainly includes the following aspects. First, it is about the research on green agriculture. In the book *Green Knowledge Shaping: Environmental Politics and Cultural Evolution*, Kniss et al. (2016) provided his fellow environmentalists with a “progressive report” on the sustainable development of human society. In *Spirit of Green Transformation*, Aroca et al. (2007) viewed the contribution of environmentalists to environmental protection and the role of green concept communication from a historical perspective. Adhikari et al. (2017) cited diffusion theory to explain the influence factors of green production adoption rate change. Milroy et al. (2013) showed that the production of green, high-quality, and safe agricultural products has been highly valued by governments and welcomed by consumers, which stimulates the green development of agriculture. Orenstein et al. (1976) defined the green development of agriculture as taking the safe production of agricultural products as the goal and emphasized that science can effectively adjust the allocation structure of agricultural production factors, so as to ensure the safe supply of agricultural products while optimizing the ecological environment at the same time. Pingali (2012) studied the influencing factors of agricultural green development. Lapple and Donnellan (2009) believed that the green development of agriculture is an exploration of the process of sustainable development of agriculture based on respect for nature, the guarantee of green development system construction and mechanism innovation, and the support of modern technology. Second is the research on the demand of funds for green agriculture. Frantál and Prousek (2016) emphasized that the importance of developing green agriculture is increasingly apparent because the development of agriculture needs enormous financial support; therefore, how to solve the problem of insufficient funds is particularly critical. Liu et al. (2020) proposed to use of green science and technology to produce green agricultural products and play a key role in financing. Pan et al. (2017) found that through the intervention measures of subsidies, taxation, public R&D, and international assistance, the green employment plan of environmental protection and recovery is formulated to promote the green development of agriculture. Raza et al. (2019) proposed to focus on the ecological compensation mechanism of agricultural green production and put forward the implementation strategy of green development policy financial support according to the actual situation of national development. Third, it is about the research on the financing behavior (FB) of farmers. Roos and Hahn (2016) used the Heckman two-stage model to analyze the main factors affecting the sample farmers’ lending behavior and credit constraints. Struik and Kuyper (2017) found that there is a linear relationship between the farmers’ own factors such as farm area, knowledge, and cultural level, and the proportion of financing they can obtain from financial institutions. Whittinghill and Rowe (2012) showed that the most important factors affecting the attitude of green farmers are health and environmental awareness. Evermann and Tate (2016) used a structural equation model to analyze the impact of age, education, and experience on farmers’ willingness to adopt organic production. Zhong (2011) used the Probit model to investigate the effects of farm characteristics, cost–benefit, and market information on organic farmers’ willingness to produce. Montoro et al. (2018) found that agricultural credit is of great significance for the development of agricultural modernization, improving farmers’ participation in the production process, and then improving production efficiency (Ponnapureddy et al., 2020). In particular, for farmers who use financing to purchase agricultural machinery and equipment, agricultural credit can also improve their net income. In the study, it is proposed that each characteristic of farmers’ economic behavior is a rational choice made by internal constraints and external environment stimulation.

The academia has formed a wealth of research results in the field of green agriculture and farmers’ FB, which has laid a solid foundation for this article to carry out the research on the financial behavior of large professional households in green agriculture, but there are still shortcomings. First, scholars are more from the traditional agricultural level to study farmers’ FB, and the research on green agriculture level needs to be supplemented. Second, the main body of the study is mainly concentrated on ordinary small farmers (Demiryurek and Ceyhan, 2008), ignoring the important impact of FB on professional large farmers as a new agricultural management body on the sustainable development of green agriculture. Third, the research on farmers’ financial behavior only stays at the national level while lacking research on farmers’ FB in provinces, especially in Heilongjiang. Fourth, the research methods lack innovation.

Based on this, the innovation of this article is mainly reflected in the following aspects. First, from the perspective of green agriculture, the FB of farmers from the level of green agricultural production is studied in this article. Second, the main body of the study is the new agricultural management subject (major professional households), and it explores the factors influencing the FB of the major households and the important role of FB in the development of green agricultural production. Third, taking Heilongjiang as the research object makes up for the defects of the past research on the FB of the farmers. Fourth, based on the theory of planning behavior, this article analyzes the influencing factors of professional large-scale FB, discusses the degree of influence of the various factors on FB by building a structural equation model, and provides innovative research methods for the study of green agriculture and FB of professional large-scale households.

## 2. Theoretical analysis framework and research hypothesis

### 2.1. Theoretical analysis framework

**FIGURE 1:**
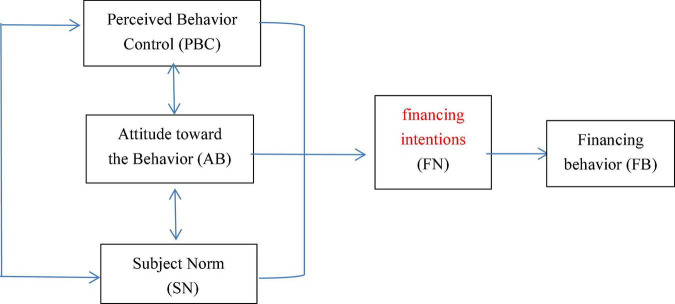
Theoretical analysis framework.

The theory of planned behavior (TPB) shows that people’s behavior is the result of careful consideration, and all the factors that may affect behavior are indirectly affected by behavior intention. For large professional households to participate in green agricultural production research, the logical relationship among the three is expressed as “financing cognition→ financing intention→ financing behavior.” In the TPB, the financing intention (FN) of large professional households to green agriculture mainly depends on three factors: Perceived behavior control (PBC), attitude toward the behavior (AB), and subjective norms (SNs). Therefore, this article analyzes the FN and FB of large professional households to participate in green agricultural production from the three aspects of PBC, AB, and SN. The theoretical framework is shown in Figure 1.

### 2.2. Research hypothesis

According to the perceived behavior control theory, when an individual thinks he or she has more resources and opportunities, he or she will expect fewer obstacles, thus the perceptual behavior control over his or her behavior will be stronger. The PBC of large professional financing can be understood as the control ability of large professional financing for green agricultural products according to their past experience and expected obstacles. Self-efficacy refers to an individual’s speculation and judgment on whether he or she has the ability to complete a certain behavior. If a large professional household has confidence in his or her financing ability, he or she will have higher self-efficacy and a positive attitude toward FB. On the contrary, he or she will have a negative attitude. If large professional households have high control and can deal with the uncertainty of green production financing according to the resources and expectations they have, they will have stronger PBC. Therefore, this article measures the PBC of large professional households on green agricultural production financing from four aspects, namely, the understanding of green agriculture and green agricultural products, the awareness of agricultural product safety and ecological protection, the ability to participate in green agricultural production financing, and the positive impact of participation in financing on green agricultural production. Based on this, hypothesis H1 is proposed.

H1: The strong PBC of large professional households has a positive impact on their FI of green agricultural production.

Attitude is formed by an individual’s evaluation of a specific behavior after conceptualization. Therefore, the composition of attitude is often regarded as a function of an individual’s significant belief in the outcome of the behavior. According to the cost–benefit theory, from an economic point of view, the expectation that benefits are greater than costs is the basic starting point of people’s behavior. For large professional households that specialize in agricultural crop planting, whether the economic utility brought about by participating in green production financing can meet the expectations is the primary principle that affects their FB and attitude, and also reflects the cognition of the consequences of FB. The subjective perception of the social and economic effects of green agricultural production and the conceptual cognition of FB are also important factors affecting their financing attitudes and behaviors. Risk preference refers to the basic attitude of individuals to take risks and is an important leading factor for individuals to perceive decision-making situations and make risk decisions. The risk attitude of large professional households toward financing green agricultural production affects their financing decisions and attitudes. The higher the risk tolerance, the greater the willingness of FB. Therefore, this article evaluates the behavior and attitude of large professional households to participate in the financing of green agricultural production from four aspects, namely, the psychological expectation of the economic effect brought by participating in the financing of green agricultural production, the cognition of the importance of ecological protection and sustainable development, the cognition of the importance of financing green agricultural production, and the risk attitude of the FB. Based on this, hypothesis H2 is proposed.

H2: The positive behavior and attitude of large professional households have a positive impact on their willingness to finance green agricultural production.

Subjective norms can be said to be the pressure that an individual and important others or groups think that whether they should take a certain behavior. The mandatory norms of large professional households on green agricultural production FB mainly come from the government while the demonstrative norms mainly come from family members, neighbors, friends, social organizations, etc. The SN of large professional households refers to the social pressure they feel when they decide whether to finance green agricultural production. Green agriculture is obviously of great significance to the sustainable development of China’s ecological environment. The promotion and publicity of green agriculture by the government can further meet the needs of agricultural producers and create greater economic benefits for farmers. The government provides corresponding subsidies, concessions, and exemptions for farmers to produce green agricultural products, which can not only effectively alleviate the pressure of agricultural financing but also form a normative concept from the perspective of social pressure. The green production concept and environmental protection awareness of the surrounding people further affect the willingness and behavior of large professional households for green agricultural production financing. SN and individual behavior intention usually show a significant positive correlation. The stronger the SN is, the stronger the FI of large professional households will be; the weaker the SN is, the weaker the FB will be. Therefore, the government’s promotion and publicity of green agriculture, the government’s preferential policies and financial subsidies, and the social atmosphere of ecological security and resource protection are used to measure the subjective normative cognition of large professional households on green production FB. Based on this, hypothesis H3 is proposed.

H3: The SNs of large professional households have a positive impact on their willingness to finance green agricultural production.

## 3. Materials and research methods

### 3.1. Data sources

Heilongjiang Province, with a green, high-quality, and high-efficiency creation demonstration county project, is an important green agriculture development base in China. In Heilongjiang Province, for example, the project of establishing green, high-quality, and high-efficiency demonstration county for rice in Jixi and Hulin is foliar fertilizer; Wudalianchi has become a national commodity grain base county, a basic farmland protection demonstration area, a national green, high-quality, and efficient demonstration county, and a national regional soybean breeding base, with an area of 1.5 million mu certified for green organic agricultural products. Baoqing County, in Shuangyashan City, declared and implemented the national green, high-quality, and efficient rice production base project. Therefore, this questionnaire conducted a field survey in 6 counties (cities) and 31 villages of 18 towns in Heilongjiang. The survey samples were determined by a stratified sampling method. The survey method mainly adopted face-to-face interviews between investigators and the major labor force of major professional households. A random sampling method was used for the questionnaire survey. A total of 287 questionnaires were issued, and 275 valid questionnaires were obtained, with an effective rate of 95.82%. The questionnaire mainly included personal information, such as gender, age, education level, family population, annual labor consumption, cultivated land area, and financing amount.

As shown in Table 1, among the 275 large professional households surveyed in this article, the number of households headed by men was 233, which exceeded the number of households headed by women. It can be seen that the surveyed large professional households are still dominated by males. From the perspective of family age, the largest population is 55–60 years old, followed by farmers who are >60 years old, and the lowest proportion of farmers is 50–55 years old. Being aged >55 years, they have the characteristics of aging, and the large aging professional households will further affect their financing willingness due to the characteristics of old thinking and risk aversion. The vast majority of farmers in the survey sample have an education level of junior high school or below, while the proportion of high school or above is very low, which indicates that the major professional households have a low level of education, and their business philosophy and financing awareness are poor. The household population accounts for the largest proportion of 3–5 people, and the annual operation consumes the labor force of at least 2–5 people. Large professional households need to meet the moderate-scale operation, and the annual operation of professional products needs to consume more than two laborers (usually hired workers). According to the survey on the area of cultivated land operated by large professional households, most farmers have an area of 100–150 mu of cultivated land, followed by 150–200 mu of cultivated land, and the proportion of cultivated land <100 mu is the lowest. In other words, the 275 large-scale professional households surveyed have a relatively large scale of cultivated land, which generally meets the requirements of large-scale professional households operating arable land of >100 mu, therefore, it is more likely for an individual to get a financing loan. According to the survey of the financing amount of large professional households, we found that the largest proportion of financing amount is <10,000:121 yuan, followed by 75 people with a financing amount of 10,000–20,000:75 yuan, and only 33 people with a financing scale of >30,000:33 yuan. This shows that professional large households are now less aware of financing, the amount of financing is small, and the conservative management method is still the choice of most large professional households.

**TABLE 1:** Investigation of sample essential information.

Index	Category	Frequency	Rate of recurrence
Gender	Male	254	92.4%
	Female	21	7.6%
Age	Under 50	45	17.7%
	50–55 years old	14	5.5%
	55–60 years old	108	42.5%
	Over 60 years old	87	34.3%
Education level	Junior high school and below	75	27.3%
	High school	99	36%
	Junior college	86	31.3%
	Undergraduate	12	4.3%
	Master degree and above	3	1.1%
Family population	<3	20	7.3%
	3–5	157	57.1%
	5–7	63	22.9%
	>7	35	12.7%
Annual labor consumption	<3	12	4.4%
	3–5	121	44%
	5–8	86	31.3%
	>8	56	20.3%
Cultivated land area	<100 mu	31	11.3%
	100–150 mu	121	44%
	150–200 mu	89	32.4%
	>200 mu	34	12.3%
Financing amount	<10 thousand	121	44%
	10–20 thousand	75	27.3%
	20–30 thousand	46	16.7%
	>30 thousand	33	12%

### 3.2. Variable selection

Based on the setting of theoretical framework, referring to the selection of relevant variables and the results of table design in the existing research, combined with field research, this article designs a total of 17 topics according to the TPB to measure the five potential variables of large professional households on green agricultural production: PBC, AB, SN, FI, and FB. The questionnaire used Likert’s five-point scale. Scores of 1–5 represent complete disagreement, disagreement, general, basic agreement, and complete agreement, respectively (Conroy and Harpe, 2015). The meaning and assignment of variables are shown in Table 2.

**TABLE 2:** Variables description and assignment.

Latent variable number	Latent variable	Observable variable
Q1	Attitude of behavior (AB)	The psychological expectation of the economic effect brought by participating in green production financing (AB1)
		Recognition of the importance of ecological protection and sustainable development (AB2)
		Recognition of the importance of green agricultural production financing (AB3)
		Risk attitude toward financing behavior (AB4)
Q2	Subject norm (SN)	Government’s promotion and publicity of green agriculture (SN1)
		Government’s preferential policies and financial subsidies (SN2)
		Social atmosphere of ecological security and resource protection (SN3)
Q3	Perceived behavioral control (PBC)	Understanding of green agriculture and green agricultural products (PBC1)
		Safety of agricultural products and awareness of ecological protection (PBC2)
		Ability to participate in green agricultural production financing (PBC3)
		Participation in financing has a positive impact on green agricultural production (PBC4)
Q4	Financing intentions (FN)	The degree of willingness to participate in green agricultural production financing (FN1)
		Willing to actively understand financing methods and financing conditions (FN2)
		Willing to help the government publicize and promote green agricultural products financing (FN3)
Q5	Financial behavior(FB)	Willing to finance under certain risk (FB1)
		Willing to improve green agricultural production technology through financing (FB2)
		Willing to achieve sustainable development of resources through financing (FB3)

Here, for convenience, we used Q1 to represent attitude of behavior, Q2 to represent subject norm, Q3 to represent perceived behavioral control, Q4 to represent financing intentions, and Q5 to represent financial behavior.

### 3.3. Research method

As an effective multivariate analysis method, structural equation modeling (SEM) has been widely used in many fields. The biggest advantage of SEM over the traditional econometric regression method is that it can deal with multiple dependent variables at the same time, allowing independent variables and dependent variables to contain measurement errors. The model can simultaneously analyze the influence of the relationship and path between the observed variables and potential variables. The specific forms of SEM constructed in this article are as follows.

Measurement equation:


(1) $$\begin{array}{c} X_{1\,i}=\beta_{j}X_{1}+e_{j}\left(i=1,2,3,4;j=1,2,3,4\right)\\ X_{2\,i}=\beta_{j}X_{2}+e_{j}\left(i=1,2,3;j=5,6,7\right)\\ X_{3\,i}=\beta_{j}X_{3}+e_{j}\left(i=1,2,3,4;j=8,9,10,11\right)\\ Y_{1\,i}=\beta_{j}Y_{1}+e_{j}\left(i=1,2,3;j=12,13,14\right)\\ Y_{2\,i}=\beta_{j}Y_{2}+e_{j}\left(i=1,2,3;j=15,16,17\right) \end{array}$$

where *X*_1*i*_,*X*_2*i*_,*X*_3*i*_,*Y*_1*i*_,*Y*_2*i*_ represent the observable variable. β_*j*_(*j* = 1,2……17) is the load factor of the observable variable. *e*_*j*_(*j* = 1,2……17) represents the residual of each regression equation.

Structural equation:


(2) $$\begin{array}{c} X_{2}=\alpha_{1}\,X_{1}+\mu_{1}\\ X_{3}=\alpha_{2}\,X_{1}+\mu_{2}\\ Y_{1}=\alpha_{3}\,X_{1}+\alpha_{4}\,X_{2}+\alpha_{5}\,X_{3}+\mu_{3}\\ Y_{2}=\alpha_{6}\,X_{1}+\alpha_{7}\,X_{2}+\alpha_{8}\,X_{3}+\mu_{4} \end{array}$$

where *X*_1_,*X*_2_,*X*_3_,*Y*_1_,*Y*_2_ represent the latent variable. α_*1*_, α_*2*_……α_*8*_ represents the path coefficient between latent variables. μ_1_,μ_2_,μ_3_,μ_4_ represents the residual between latent variables.

## 4. Results and analysis

### 4.1. Reliability analysis

The reliability test refers to the reliability of the questionnaire. It refers to the repeated measurement of the same object by the same method. The credibility of the questionnaire is also the reliability of the questionnaire, which refers to the consistency of the results obtained when the same method is used to repeatedly measure the same object, that is, the degree of reflecting the actual situation. The most commonly used measurement index is Cronbach’s alpha coefficient. It is generally considered that 0.60–0.65 is unacceptable, 0.65–0.70 is the minimum acceptable value, 0.70–0.80 is better, and 0.80–0.90 is very good (Table 3). Cronbach’s alpha of the questionnaire is 0.900, which belongs to high reliability. The overall Cronbach’s alpha coefficients of AB, SN, PBC, FI, and FB are 0.902, 0.888, 0.914, 0.946, and 0.871, respectively. The consistency and stability of the questionnaire are very good.

**TABLE 3:** Reliability statistics.

Project	Cronbach’ alpha	Number of items
All measured variables	0.900	17
Behavioral attitude measurement variables	0.902	4
Subjective norm measurement variables	0.888	3
Perceptual behavioral control measurement variables	0.914	4
Measurement variables of financing intentions	0.946	3
Measurement variables of financing behavior	0.871	3

### 4.2. Validity test

Validity analysis, simply speaking, is the validity and accuracy of the questionnaire design, which is used to measure whether the item design is reasonable. Validity can be divided into validity, construct validity, and criterion validity. Construct validity refers to the corresponding relationship between measurement items and measurement dimensions. There are two measurement methods, namely, exploratory factor analysis and confirmatory factor analysis. An exploratory factor analysis is the most widely used method to measure construct validity. In order to further measure whether the overall structure of the questionnaire is reasonable or not, exploratory factor analysis is carried out on latent variables. The exploratory factor analysis is a technique used to find out the essential structure of multivariate observation variables and reduce the dimension. First, this article tests the consistency of variables. By using SPSS 24.0 and the principal component extraction method, it uses the maximum variance rotation method to analyze the data. The results showed that the value of Kaiser–Meyer–Olkin (KMO) was 0.835 (as shown in Table 4), the Bartlett spherical test value was 0.000, and the test results were significant. These data were suitable for factor analysis. After the orthogonal rotation, the observed variables were aggregated into five components; this is the same as the original components and consistent with the theoretical model. The total cumulative variance of the five common factors is 82.207% (as shown in Table 5), indicating that the potential scalar design is reasonable and the scale has good structural validity. It is generally assumed that the value of factor load >0.5 indicates a higher convergence of the scale (Table 6). Among the factors to which the questionnaire belongs, the more the factor load value of measurement variables is >0.5, the higher the discriminant validity between the factors. After calculation, the factor load values of 17 of the five potential variables were >0.5, indicating that the questionnaire has good convergence validity and discriminant validity. AB, PBC, SN, FI, and FB have good explanatory power.

**TABLE 4:** Kaiser–Meyer–Olkin and Bartlett test results.

Kaiser-Meyer-Olkin		0.835
Bartlett sphericity test	Approximate chi-square	2610.705
	Freedom	361
	Significance	0.000

**TABLE 5:** Principal component analysis was used to extract the results.

Component	Extract the load sum of squares			Sum of squares of rotational loads		
	Total	Variance percentage	Cumulative percentage	Total	Variance percentage	Cumulative percentage
1	6.671	39.243	39.243	3.213	18.898	18.898
2	2.098	12.343	51.585	3.131	18.419	37.318
3	2.055	12.085	63.670	2.646	15.562	52.880
4	1.790	10.528	74.198	2.541	14.945	67.825
5	1.362	8.009	82.207	2.445	14.382	82.207

**TABLE 6:** Extraction factor.

	Component				
	1	2	3	4	5
AB1		0.849			
AB2		0.876			
AB3		0.848			
AB4		0.779			
SN1				0.877	
SN2				0.870	
SN3				0.859	
PBC1	0.847				
PBC2	0.847				
PBC3	0.889				
PBC4	0.834				
FN1			0.882		
FN2			0.845		
FN3			0.869		
FB1					0.870
FB2					0.883
FB3					0.858

### 4.3. Model fitness analysis

One of the hypotheses of the SEM model is that the expected covariance matrix minus the sample covariance matrix is 0. According to this hypothesis, the fitness index is proposed and divided into two categories: One is to check whether the similarity of the two models is the highest (the highest value is 1, >0.8 is acceptable, >0.9 is good), the other is to check whether the dissimilarity of the two models is the minimum (the minimum is 0, ≤0.8 is acceptable, <0.5 is good). The first type is the benign fitness index (GFI), the comparative fitness index (CFI), the regulatory fitness index (NFI), the relative fitness index (RFI), and the value-added fitness index (IFI), in which GFI = 0.902, NFI = 0.923, CFI = 0.963, RFI = 0.906, and IFI = 0.963. The second type is the root-mean-square error of approximation (RMSEA) = 0.066.

As shown in Table 7, the value of the chi-square degree of freedom ratio (CMIN/DF) is 1.856, which conforms to the fitting standard (1 < CMIN/DF < 3), and indicates that the sample data have a good degree of fit with the theoretical model, and the comprehensive fitting degree is good. Because the chi-square degree of freedom of SEM is easily affected by the sample size, and the SEM fitting criterion is not unitary, the evaluation index should be objective and comprehensive. In the absolute fitting index, GFI = 0.902 > 0.9.

**TABLE 7:** Model fitting evaluation standard and evaluation index.

Index	Model fitting value	Fitting standard	Judgment result
CMIN/DF (chi-square degree of freedom ratio)	1.856	1<CMIN/DF<3	Good fitness
GFI (benign fitness index)	0.902	GFI>0.9	Good fitness
RMSEA (root-mean-square error of approximation)	0.066	RMSEA<0.08	Reasonable fitness
NFI (regulatory fitness index)	0.923	NFI>0.9	Good fitness
CFI (comparative fitness index)	0.963	CFI>0.9	Good fitness
RFI (relative fitness index)	0.906	RFI>0.9	Good fitness
IFI (value-added fitness index)	0.963	IFI>0.9	Good fitness

Root-mean-square error of approximation = 0.066 (<0.08), which indicates that the absolute fitting degree of the model is high, and the indexes are well matched. NFI = 0.923 (>0.9), CFI = 0.963 (>0.9), RFI = 0.906 (>0.9), and IFI = 0.963 (>0.9), which are all ≥0.9, indicating that the model has good value-added fitting. In total, it can be seen that the model as a whole meets the fit standard, and the fitness degree of the structural equation is very good.

### 4.4. Analysis of model results

#### 4.4.1. Mediating effect test

To further explore the influencing factors of large professional households’ participation in green agricultural production FB, this article uses AMOS25.0 software to carry out the BOOTSTRAP test to explore its mediating effect. The random sample is set at 2,000, and the confidence interval is 95%. The results are shown in Table 8.

**TABLE 8:** Mediating effect test results.

Route	Standardized path coefficient	Bootstrap	
		Lower	Upper
AB→FN→FB	0.177	0.072	0.347
PBC→FN→FB	0.184	0.085	0.340
SN→FN→FB	0.200	0.092	0.375

The confidence interval of “AB→FN→FB” is (0.072, 0.347), and the intermediate effect coefficient is 0.177. The confidence interval of “PBC→FN→FB” is (0.085, 0.340), and the intermediate effect coefficient is 0.184. The confidence interval of “SN→FN→FB” is (0.092, 0.375), and the intermediate effect coefficient is 0.200. Both ends of the confidence interval of the three paths are positive, excluding 0. Therefore, the FN has a significant intermediary effect on the participation of family farmers in the FB of green agricultural production.

#### 4.4.2. Results analysis

**FIGURE 2:**
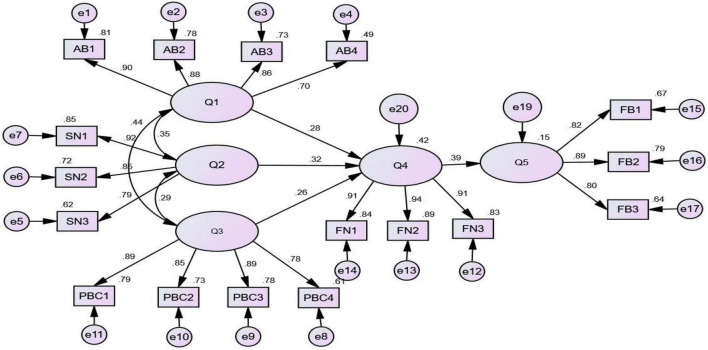
Path coefficient diagram about the influence of large professional households on FN and FB of green agricultural production.

According to the research model, after running AMOS25.0 software, the SEM and standardized path chart of large professional households on FN of green agricultural production were obtained. As shown in Figure 2, the path coefficient of each latent variable of the model is significant at 1% of the significance level, which has passed the test. It shows that the AB, PBC, and SN of large professional households have a significant impact on their FN to participate in green agricultural production (Table 9). The specific analysis is as follows:

**TABLE 9:** Path analysis results.

	Standardization coefficient	CR	*P*-value
FN←AB	0.320	3.872	***
FN←PBC	0.403	4.650	***
FN←SN	0.337	3.655	***
FB←FN	0.361	5.175	***

***Remarkable.

(1) The path coefficient of PBC on the FN of large professional households for green agriculture production is 0.258, and their PBC has a significant impact on their FN, indicating that the fewer obstacles that large professional households expect based on past experience and the more resources they have, the stronger FN of large professional households will be. That is, hypothesis H1 holds: The strong PBC of large professional households has a positive impact on their FN of green agricultural production. From the perspective of each observation variable, the degree of understanding of green agriculture and green agricultural products (PBC1) contributed the most to the positive impact of PBC (the standardized coefficient was 0.887), followed by the ability to participate in green agricultural production financing (PBC3, the standardized coefficient was 0.886), then the awareness of agricultural product safety and ecological protection (PBC2, the standardized coefficient was 0.855), and, finally, the participation in financing has a positive impact on green agricultural production (PBC4, standardization coefficient is 0.782). Most of the major professional households are risk-averse. Only when they feel that the income generated by green agricultural production financing is greater than its financing cost, they can produce positive PBC. In addition, their own conditions and financing ability are also important factors. On one hand, if farmers are older and less educated, then their understanding of the importance of financing green agricultural production is lower, which will affect their FN and FB. On the other hand, the long-term environment, financing atmosphere, and ecological protection awareness of large professional households need not only their own initiative to improve but also need the government to implement accurate and strong policy publicity and financing support. It also needs to strengthen the dissemination and training of financial knowledge of large professional households.
(2) The path coefficient of AB on the FN of large professional households is 0.277, which has passed the 1% significance level test, and there is a positive correlation between them, indicating that the more positive the AB of large professional households is, the stronger their FN is, that is, hypothesis H2 holds: The positive AB of large professional households has a positive impact on their FN for green agricultural production. From the perspective of each observation variable, the psychological expectation of economic utility brought by participating in green production financing (AB1) has the largest contribution to AB (the standardized coefficient is 0.902), followed by the cognition of the importance of ecological protection and sustainable development (AB2, the standardized coefficient is 0.882), then the cognition of the importance of green agricultural production financing (AB3, the standardized coefficient is 0.855), and, finally, the risk attitude toward FB (AB4, standardized coefficient is 0.703). In this article, trust is regarded as a kind of “psychological expectation” that will have an impact on the public’s behavior and decision-making. This is also supported by the conclusion of our survey of 275 large professional households. On one hand, the psychological expectation of large professional households for the economic benefits brought by their participation in green production financing comes from the cognition of their own operation and production capacity. On the other hand, it is the issue of trust in the implementation of policies by the government, that is, whether the publicity measures, compensation, and financing preferences can be effectively implemented.
(3) The path coefficient of SN on the FN of large professional households is 0.321, which has passed the 1% significance level test, and there is a positive correlation between them. SNs have the most significant impact on the FN of large professional households in green agricultural production than PBC and AB, indicating that if the large professional households face greater social pressure on whether to carry out the FB of green agricultural production, they will have a stronger FN, which means the hypothesis H3 holds: the SNs of large professional households have a positive impact on their FN for green agricultural production. From the observation variables, the government’s promotion and publicity of green agriculture (SN1) have the greatest positive impact on SN (standardization coefficient is 0.921), followed by the government’s preferential policies and financial subsidies (SN2, standardization coefficient is 0.849), and, finally, the social atmosphere of ecological security and resource protection (SN3, standardization coefficient is 0.787). From this, we can see that the government’s mandatory norms have a greater driving effect on the FN of large professional households to participate in green agricultural production than the model norms, which also shows that their SNs to participate in green agricultural production financing are largely affected by the government’s efforts to promote and publicize green agriculture, as well as the government’s preferential policies and financial subsidies. It also makes large professional households show awe and obedience to the policies implemented by the government. This top-down influence on large professional households makes the government play a very good leading role.

## 5. Conclusion and policy implications

As an important starting point of agricultural moderate-scale operation and development of modern agriculture, large professional households are motivated to actively raise funds to promote green agriculture production, which is an inevitable choice for China to develop modern green agriculture. It is also of great significance to the security of the agricultural ecosystem, national food security, and the promotion of agricultural international market competitiveness. Based on the TPB, this article investigates 275 professional households in 6 counties (cities), 18 townships, and 31 villages of Wudalianchi City, Hulin City, Baoqing County, Ning’an City, Jixi City, and Hegang Suibin County in Heilongjiang as research samples. By directional designing, distributing, collecting, and sorting out questionnaires, based on the SEM, this article investigates the factors influencing the FB of large professional households and green agricultural production in Heilongjiang, and concludes that the AB, PBC, and SN of large professional households have a significant impact on their FN of green agricultural production, which can further affect the FB of large professional households. Therefore, the following countermeasures are put forward:

The main conclusions are as follows. (1) From the perspective of perceptual behavior control, we found that the financing willingness of large professional households is affected by past experience expectations, resources at their disposal, their understanding of green agricultural products, and their participation in green agricultural production. Financing ability is an important factor affecting financing willingness and FB. (2) From the perspective of behavioral attitudes, the psychological expectations of the economic utility brought by the participation of large professional households in green agricultural production financing greatly affect their financing willingness, which, in turn, affects their FB’s. From the perspective of SNs, the social pressure felt by large professional households on whether to finance green agricultural production has the most significant impact on their willingness to finance. The following countermeasures are therefore proposed:

(1) Strengthening the construction of rural talents and improving the training system for new professional farmers is the key. At present, the large professional households in Heilongjiang Province have problems of serious aging and generally low education level. In order to improve the production and operation knowledge and concepts, financial product recognition, and scientific and cultural literacy of large professional households, the local government and educational institutions in Heilongjiang Province should focus on promoting rural areas. Build a talent training system, establish a comprehensive talent assessment and incentive mechanism, and at the same time strengthen the introduction of talents with advanced scientific research conditions and high-quality, and expand the professional and large-scale talent team.
(2) Improve the production and operation capabilities of large professional households, and strengthen the cultivation of green agricultural producers’ moral culture, market awareness, and management skills. At the same time, the government should effectively implement financial support for large professional households engaged in green agricultural production. In order to strengthen the awareness of ecological protection and sustainable development of major professional households, and understand the importance of financing for green agricultural production, the government should promote the new pattern of “green product” modern agricultural development, pay attention to the publicity work on ecological protection and sustainable development, and improve professional large-scale households. The awareness of the importance of developing green agricultural products enables large professional households to truly understand the connotation of the concept of “strong agriculture, beautiful rural areas, and rich farmers” and creates a good ideological foundation and a social atmosphere for active participation in financing for large professional households in green production financing.
(3) Strengthen the promotion and publicity of green agriculture. The government should promote the new pattern of “green product” modern agricultural development, pay attention to the publicity of ecological protection and sustainable development, raise awareness of the importance of developing green agricultural products among major professional households, and truly understand “strong agriculture, beautiful rural areas, and rich farmers” concept meaning. Second, the government should effectively implement preferential policies and financial subsidies for large professional households to participate in green agricultural production, expand financing channels, accelerate the development of small- and medium-sized rural financial systems, and develop new types of rural financial institutions such as rural cooperatives, village banks, and loan companies. Credit cooperatives in the core position should actively implement the strategy of strategic transformation, increase financial support, invest a certain amount of funds as special funds for the development of large professional households engaged in green agricultural production, and promote the revitalization of rural green industries. Finally, the government should focus on developing a social atmosphere of ecological security and resource protection, speed up the development of the green agricultural value chain, and further develop the industrialized management of green agriculture. In order to promote the development plan of green industry and improve the production efficiency of green agricultural products for professional large-scale households, the Heilongjiang Provincial Government should focus on promoting the construction of marketing channels for green agricultural products, promoting the new online “Internet+” marketing method, and developing online e-commerce platforms and green agricultural products trading display The platform creates a good ideological foundation and a social atmosphere for actively participating in financing for large professional green production financing.

## Data availability statement

The original contributions presented in this study are included in the article/supplementary material, further inquiries can be directed to the corresponding author.

## Ethics statement

Ethical review and approval was not required for the study on human participants in accordance with the local legislation and institutional requirements. Written informed consent from the (patients/participants OR patients/participants legal guardian/next of kin) was not required to participate in this study in accordance with the national legislation and the institutional requirements.

## Author contributions

SZ: conceptualization, methodology, and software. XX: data curation and writing—original draft preparation. JL: writing—reviewing and supervision. NW: software. All authors contributed to the article and approved the submitted version.
